# Stent Geometry Matters: Impact of Tapered *vs.* Straight Stents On Persistent Hemodynamic Depression After Carotid Artery Stenting

**DOI:** 10.2174/0115734056439426260326041814

**Published:** 2026-04-08

**Authors:** Mustafa Demir, Yunus Yasar, Abdulbaki Agackiran, Emine Yilmaz, Aslihan Semiz-Oysu

**Affiliations:** 1 Department of Radiology, University of Health Sciences, Umraniye Training and Research Hospital, Istanbul, Türkiye; 2 Department of Radiology, Bahçeşehir School of Medicine, Istanbul, Türkiye; 3 Department of Neurology, University of Health Sciences, Umraniye Training and Research Hospital, Istanbul, Türkiye; 4 Department of Radiology, Marmara University School of Medicine, Istanbul, Türkiye

**Keywords:** Carotid artery stenting, Hemodynamic depression, Stent design, Carotid sinus, Angiography, Tapered stents

## Abstract

**Introduction:**

Carotid Artery Stenting (CAS) is an established alternative to carotid endarterectomy, particularly in patients with elevated surgical risk. Hemodynamic Depression (HD) is a common complication that is usually transient; however, persistent HD may compromise cerebral perfusion. The specific impact of stent geometry on persistent HD remains underexplored.

**Methods:**

This retrospective study included 109 patients who underwent primary CAS between October 2020 and October 2023. HD was defined as intraoperative hypotension (systolic blood pressure < 90 mmHg) and/or bradycardia (heart rate < 60 bpm), while persistent HD was defined as lasting more than 24 hours despite medical therapy. Patients were evaluated according to stent geometry (straight *vs.* tapered), carotid bulb involvement, plaque characteristics, and procedural variables. Multivariable logistic regression was performed, adjusting for demographic, clinical, anatomical, and procedural factors.

**Results:**

Periprocedural HD occurred in 40.4% of patients, and persistent HD in 25.7%. Persistent HD was significantly more frequent in patients treated with straight stents than in those treated with tapered stents (39.5% *vs.* 18.6%). Carotid bulb involvement and the use of a straight stent were significantly associated with persistent HD. In multivariable analysis, straight stent use (OR: 1.446; 95% CI: 1.041-2.018) and carotid bulb involvement (OR: 1.490; 95% CI: 1.102-2.015) remained independently associated with persistent HD. Twelve-month clinical outcomes did not differ between stent groups.

**Discussion:**

Persistent hemodynamic depression after carotid artery stenting appears to be influenced not only by patient-related factors but also by procedural characteristics, particularly stent geometry and carotid bulb anatomy. The independent association of straight stent use with prolonged hemodynamic instability underscores the clinical relevance of device selection beyond technical success. Incorporating anatomical conformity into preprocedural planning may reduce prolonged baroreceptor-mediated instability and improve periprocedural safety.

**Conclusion:**

Persistent HD is a clinically relevant complication after CAS. Individualized stent selection may help reduce prolonged hemodynamic instability.

## 1 INTRODUCTION

Carotid artery stenosis is a significant risk factor for ischemic stroke and Transient Ischemic Attacks (TIA), significantly contributing to global morbidity and mortality rates [1-3]. While Carotid Endarterectomy (CEA) has long been considered the standard treatment, Carotid Artery Stenting (CAS) has emerged as a less invasive alternative, particularly for patients with high surgical risk [4, 5]. However, compared to open surgical procedures like CEA, CAS is more prone to complications such as Hemodynamic Depression (HD) due to mechanical stimulation of the carotid sinus during the intervention.

HD, typically characterized by intraoperative hypotension and bradycardia, may compromise cerebral perfusion and directly affect procedural safety and patient prognosis [6-9]. Persistent HD-defined as bradycardia and/or hypotension lasting beyond 24 hours despite medical therapy-has been linked to adverse outcomes in some studies, but reported rates are heterogeneous, ranging between approximately 11% and 35% depending on definitions; for instance, a retrospective series found persistent hypotension occurred in about 11-35% of cases post-CAS, depending on the criteria used [10, 11].

The shape and mechanical properties of the stents used during CAS are essential determinants of the pressure exerted on the carotid sinus. Straight self-expanding stents may not conform to the natural anatomy of the carotid artery, particularly in cases with marked diameter mismatch between the Common Carotid Artery (CCA) and the Internal Carotid Artery (ICA) [12-14]. This mismatch can result in excessive radial force on the ICA level and trigger baroreceptor-mediated hemodynamic responses [6]. In contrast, tapered stents, with their gradually narrowing design toward the distal segment, may better accommodate the natural vascular taper and reduce mechanical stress on the carotid sinus, potentially lowering the risk of HD. While tapered stents have been hypothesized to reduce carotid sinus stimulation by improving anatomical conformity, comparative clinical data supporting this hypothesis are limited [9, 15].

Although several studies have investigated the risk factors and clinical consequences of HD following CAS, the impact of stent design on this pathophysiological process remains poorly defined [15]. Furthermore, while CAS has increasingly been preferred over CEA in selected patients, newer alternatives, such as Transcarotid Artery Revascularization (TCAR), are gaining attention; however, our study focuses explicitly on conventional CAS.

This study aims to compare the incidence of intraoperative and persistent HD between tapered and straight stents used in CAS and to determine anatomical and procedural predictors. We hypothesize that stent geometry and carotid bulb involvement are independent predictors of persistent HD.

## 2 MATERIALS AND METHODS

### 2.1 Study Design and Patient Selection

**Fig. (1):**
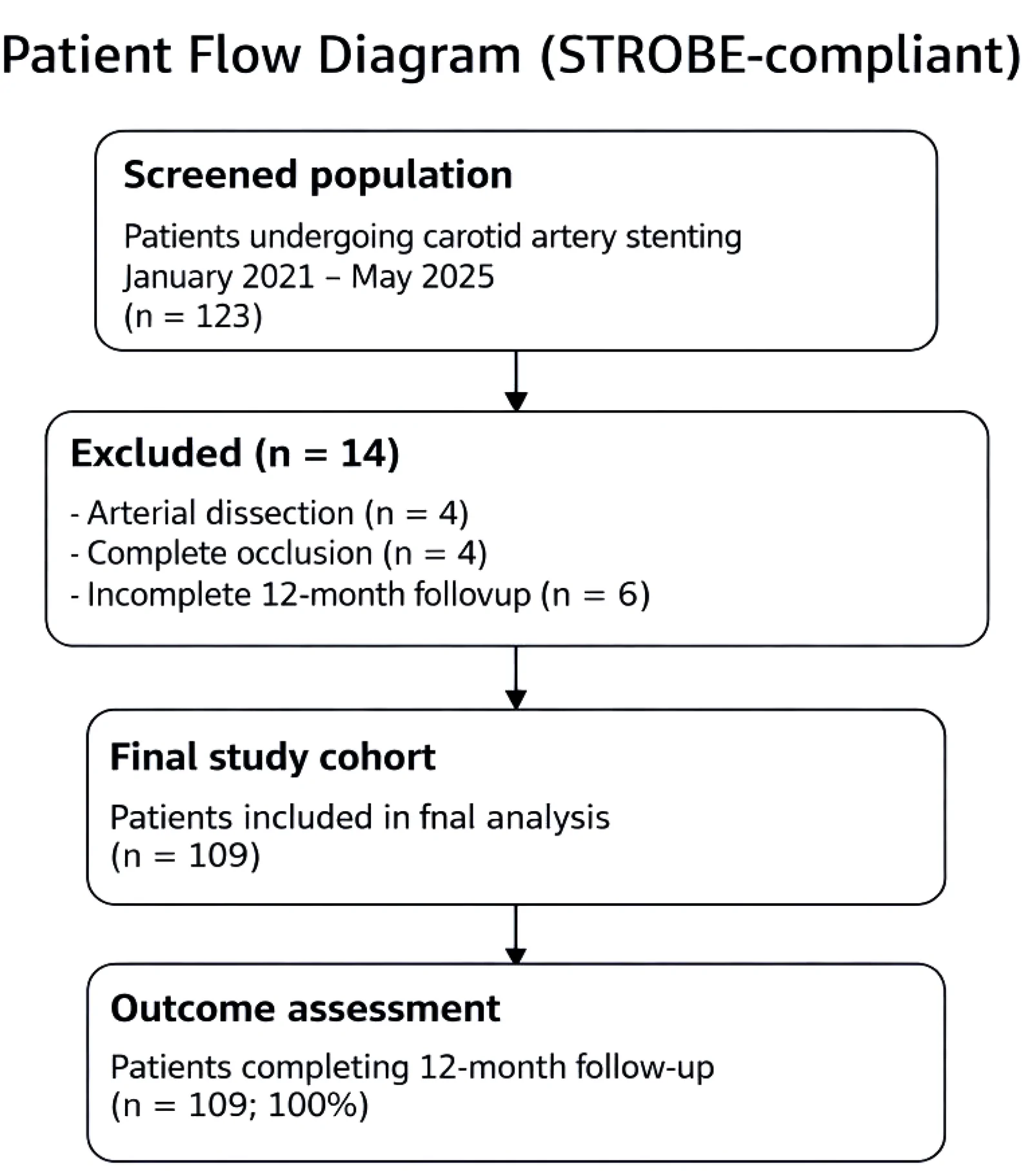
Flowchart illustrating patient selection and exclusion criteria for the study. A total of 123 patients underwent carotid artery stenting; 14 were excluded due to arterial dissection, complete occlusion, or lack of follow-up data, leaving 109 patients in the final analysis.

This retrospective study was conducted at a tertiary referral center between October 2020 and October 2023. A total of 109 consecutive patients who underwent elective CAS for internal carotid artery stenosis were included. Inclusion criteria were symptomatic carotid artery stenosis ≥ 50% or asymptomatic stenosis ≥ 70%, in accordance with contemporary clinical practice guidelines, including the Society for Vascular Surgery clinical practice guidelines for the management of extracranial cerebrovascular disease [13]. At our institution, carotid artery stenting was recommended for patients who were considered anatomically and technically suitable for the procedure, based on multidisciplinary assessment, rather than being limited exclusively to patients categorized as high surgical risk for carotid endarterectomy. Exclusion criteria were emergency procedures, tandem lesions requiring intracranial intervention, restenosis after previous CAS or CEA, and incomplete follow-up data (Fig. **1**). Patients with incomplete clinical or follow-up data were excluded from the study before analysis. The final study population consisted of 109 consecutive patients who met all inclusion criteria and had complete follow-up data during the study period. No *a priori* sample size or power calculation was performed, as this was a retrospective exploratory study. During the study period, 123 patients underwent carotid artery stenting. Eight patients were excluded due to arterial dissection or complete carotid artery occlusion. Of the remaining patients, six did not complete the 12-month follow-up and were excluded. The final study population comprised 109 patients, all of whom completed a 12-month clinical follow-up. All procedures were performed by two experienced interventional radiologists, each with more than 10 years of practice in neurointerventional procedures. The institutional ethics committee approved this retrospective study. Due to the study's retrospective nature, the requirement for written informed consent was waived. All procedures were conducted in accordance with the principles of the Declaration of Helsinki.

All consecutive patients who met the inclusion criteria and completed the predefined follow-up period were included to maximize the available sample and reflect real-world clinical practice. The number of eligible cases, therefore, determined the sample size during the study period rather than by formal power estimation.

### 2.2 Definition of Hemodynamic Depression

Hemodynamic Depression (HD) was defined as intraoperative bradycardia (heart rate < 60 bpm) and/or hypotension (systolic blood pressure < 90 mmHg) occurring during or immediately after stent deployment. Persistent HD was defined as HD lasting > 24 hours despite medical therapy and requiring vasopressor support or temporary pacing when necessary. Patients were categorized into three groups: no HD, transient HD, and persistent HD.

### 2.3 Imaging and Procedural Details

All patients underwent Digital Subtraction Angiography (DSA) before the procedure to confirm stenosis severity and vascular anatomy. Cross-sectional imaging (CTA or MRA) was used in 87% of cases to complement vessel measurements and stent planning. Stenosis severity was calculated according to the North American Symptomatic Carotid Endarterectomy Trial (NASCET) criteria. Lesions were classified as involving or not involving the carotid bulb. Plaque morphology (calcified *vs.* non-calcified) was assessed on CTA/MRA and DSA.

Carotid Artery Stenting (CAS) was performed under local anesthesia in all patients. Embolic protection devices were used in all cases. Predilatation was performed using a 3-4 mm balloon, followed by stent deployment (straight or tapered self-expanding nitinol stents, according to operator preference and vessel geometry) Fig. (**2**). Stent classification was based on manufacturer-defined design characteristics: tapered stents were conical Protégé stents (Medtronic), featuring a proximal-to-distal diameter reduction, whereas straight stents were uniform-diameter Wallstents (Boston Scientific). All stents were intentionally deployed to cover the carotid bulb, with proximal extension into the common carotid artery, in accordance with standard clinical practice. Postdilatation was selectively performed when residual stenosis exceeded 30%. Procedural parameters (predilatation and postdilatation balloon diameter, stent diameter, ICA radius) were recorded.

### 2.4 Perioperative Management

All patients received dual antiplatelet therapy (aspirin 100 mg and clopidogrel 75 mg) for at least 5 days before CAS, or received a loading dose on the day before the procedure. Dual antiplatelet therapy was continued for at least 3 months post-procedure, followed by lifelong aspirin. Periprocedural anticoagulation with unfractionated heparin was administered to achieve an Activated Clotting Time (ACT) of 250-300 seconds.

Blood pressure and heart rate were monitored continuously during the procedure and for at least 48 hours post-procedure. Patients with persistent HD were managed with intravenous fluids, vasopressor infusion, and atropine as needed. Temporary pacing was available for refractory cases, although not required in this series.

### 2.5 Outcomes and Follow-Up

The primary outcome was persistent HD. Secondary outcomes included periprocedural complications (stroke, TIA, myocardial infarction, access-site complications) and 12-month clinical and imaging follow-up (restenosis, occlusion, recurrent stroke/TIA, mortality). Carotid duplex ultrasonography was performed at 1, 6, and 12 months, supplemented with CTA when restenosis was suspected. Outcome assessment was not blinded to stent type, as stent geometry was inherently identifiable on angiographic imaging. Hemodynamic outcomes were evaluated using objective physiological parameters, including heart rate and blood pressure, recorded through standardized intraoperative and postoperative monitoring protocols.

### 2.6 Statistical Analysis

Continuous variables were expressed as mean ± SD and compared using Student’s t-test or Mann-Whitney U test, as appropriate. Categorical variables were presented as counts and percentages, and compared using chi-square or Fisher’s exact test. Variables with *p* < 0.10 in univariate analysis were entered into a binary logistic regression model to identify independent predictors of persistent HD. Odds Ratios (OR) with 95% Confidence Intervals (CI) were calculated. Statistical analyses were performed using SPSS version 26.0 (IBM Corp., Armonk, NY, USA). A two-tailed *p* < 0.05 was considered statistically significant.

## 3 RESULT

### 3.1 Baseline Characteristics According to the Presence of Hemodynamic Depression

Of the 109 patients included, periprocedural Hemodynamic Depression (HD) occurred in 44 patients (40.4%). Among these, 28 patients (25.7% of the overall cohort, 63.6% of those with periprocedural HD) experienced persistent HD, defined as hemodynamic instability lasting more than 24 hours despite medical therapy.

There were no statistically significant differences between HD and non-HD patients in terms of age, sex, hypertension, diabetes mellitus, coronary artery disease, smoking history, symptomatic stenosis, or previous stroke (all *p* > 0.05). Similarly, no baseline clinical characteristics were significantly different between patients with and without persistent HD. The mean age was 71.3 ± 6.1 years in the HD group, 71.0 ± 6.7 years in the non-HD group, and 71.1 ± 7.0 years among those with persistent HD (*p* > 0.05). The proportion of female patients was also similar across groups (HD: 24.0%, non-HD: 26.0%, persistent HD: 21.4%) (Table **1**).

### 3.2 Anatomical and Procedural Characteristics

Carotid bulb involvement was significantly more common among patients with HD (79.5% *vs.* 61.5%; *p* = 0.047) and those with persistent HD (96.4% *vs.* 56.8%; *p* < 0.001). Calcified plaques were also significantly more frequent in patients with intraoperative HD (27.3% *vs.* 90.8%; *p* < 0.001) and those with persistent HD (25.0% *vs.* 77.8%; *p* < 0.001), suggesting a possible association between plaque morphology and hemodynamic instability.

There were no statistically significant differences in pre- or postdilatation balloon diameters between patients with and without periprocedural or persistent HD. The mean predilatation balloon diameter was 3.3 ± 0.6 mm in the HD group and 3.1 ± 0.8 mm in the non-HD group (*p* = 0.103). Similarly, the mean postdilatation balloon diameter was 6.1 ± 0.4 mm in the HD group and 5.8 ± 0.9 mm in the non-HD group (*p* = 0.287). Among patients with persistent HD, the corresponding predilatation and postdilatation balloon diameters were 3.7 ± 0.8 mm and 6.2 ± 0.7 mm, respectively, which were not significantly different from those without persistent HD (*p* = 0.873 and *p* = 0.521, respectively). Stent diameter, preoperative blood pressure, and heart rate also did not differ significantly between groups (all *p* > 0.05) (Table **1**).

### 3.3 Hemodynamic Outcomes According to Stent Type

When patients were grouped by stent type, intraoperative HD was observed in 50.0% of those receiving Straight Cell Stents (SCS) and 35.7% of those receiving Tapered Cell Stents (TCS), with the difference statistically significant (*p* < 0.001). Persistent HD was also significantly more frequent in the SCS group (39.5%) compared to the TCS group (18.6%) (*p* < 0.001). Persistent hemodynamic depression was significantly more frequent in the straight stent group compared with the tapered stent group (39.5% *vs.* 18.6%), corresponding to a relative risk of 2.13 (95% CI: 1.14-4.00). Bradycardia and hypotension patterns are shown in Table **2**.

### 3.4 Regression Analysis

A binary logistic regression analysis was conducted to identify independent predictors of persistent HD. The variables included in the model-stent type, carotid bulb lesion, and calcified plaque-were selected based on univariate analysis (*p* < 0.1) and prior literature. The use of a straight stent was found to be an independent predictor of persistent HD (OR: 1.446; 95% CI: 1.041-2.018; *p* = 0.030), along with the presence of a carotid bulb lesion (OR: 1.490; 95% CI: 1.102-2.015; *p* = 0.010). The presence of calcified plaque (OR: 1.345; 95% CI: 0.642-5.259; *p* = 0.670) was not significantly associated with persistent HD in the final model (Table **3**).

### 3.5 Complications and 12-Month Outcomes

During the 12-month follow-up period, the incidence of significant complications, such as stroke, TIA, restenosis, stent occlusion, myocardial infarction, pseudoaneurysm/hematoma, and mortality, did not differ significantly between the TCS and SCS groups (all *p* > 0.05) (Table **4**). All patients were maintained on dual antiplatelet therapy, and the overall clinical course was similar between groups. Patients with persistent HD, however, required closer hemodynamic monitoring and supportive treatment during the early postoperative period.

## 4 DISCUSSION

**Fig. (3):**
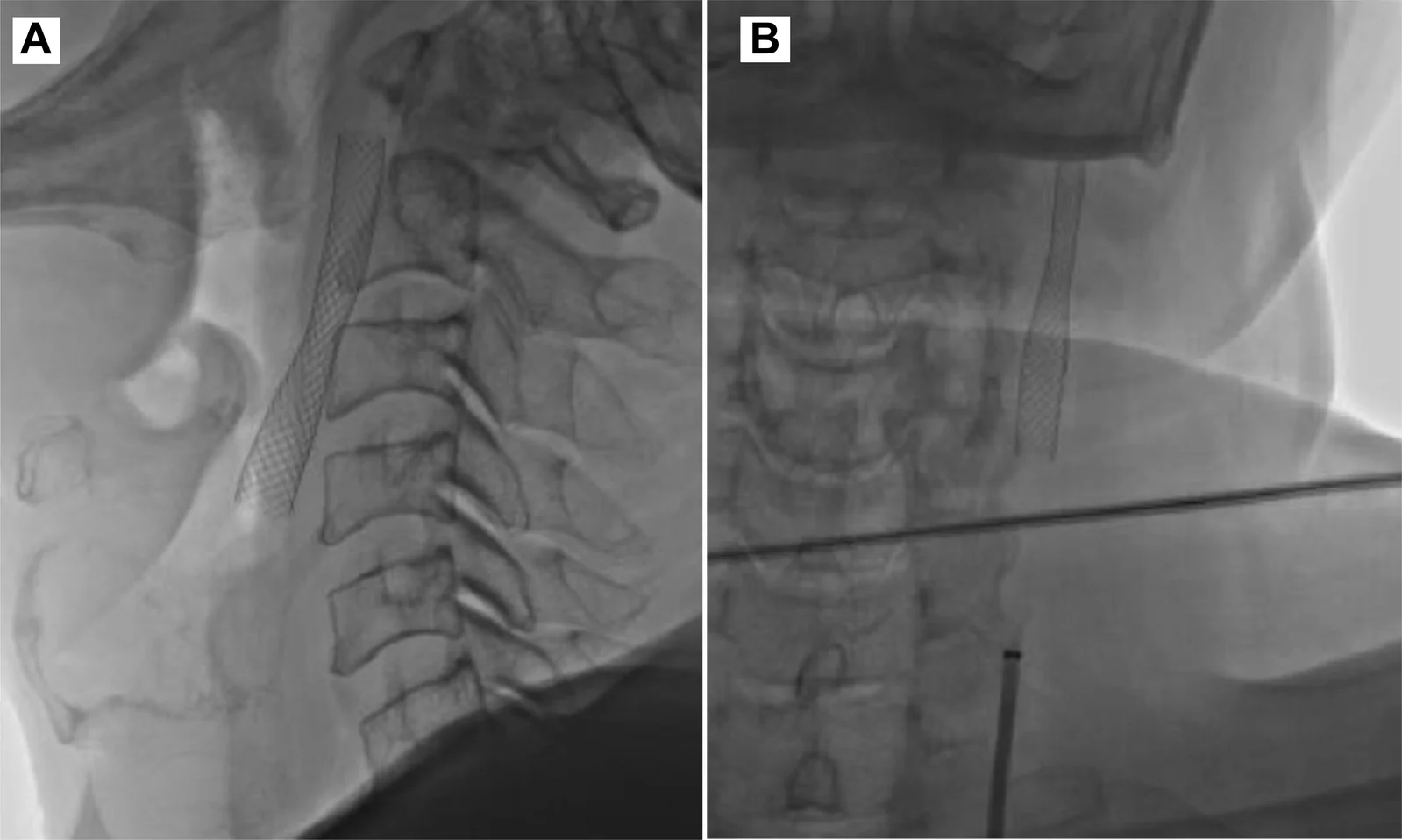
Fluoroscopic images demonstrating the design differences between straight and tapered carotid stents. **A** shows a straight stent with a uniform diameter, which may exert increased radial force at the internal carotid artery due to vessel size mismatch. **B** illustrates a tapered stent with a gradually narrowing profile toward the distal segment, offering better conformity to the native vascular anatomy.

While CEA remains the preferred treatment for patients at low to standard surgical risk, CAS represents an established alternative in selected patients with elevated surgical risk or unfavorable anatomical features. A key limitation of CAS is the risk of HD, typically triggered by mechanical stimulation of the carotid sinus. Persistent HD-defined as bradycardia and/or hypotension lasting more than 24 hours despite medical therapy-has been linked to impaired cerebral perfusion and adverse clinical outcomes [6, 8]. While numerous studies have examined HD's overall incidence, risk factors, and consequences, few have specifically addressed persistent HD. Moreover, the potential influence of procedural and anatomical factors such as stent design-particularly straight versus tapered stents-remains underexplored (Fig. **3**). The existing literature often involves heterogeneous cohorts and overlooks procedural nuances, such as stent conformity [15-17].

While several studies have investigated hemodynamic depression following carotid artery stenting, most have primarily focused on transient or periprocedural hemodynamic events. Evidence specifically examining the relationship between stent geometry-particularly tapered versus straight stent designs-and persistent hemodynamic depression remains limited. Notably, only a small number of studies, such as that by Csobay-Novák *et al*., have directly addressed the impact of stent selection on persisting hemodynamic depression [16].

This study focuses on persistent HD as a primary endpoint and evaluates contributing procedural and anatomical variables, including stent geometry, carotid bulb involvement, plaque morphology, and postdilatation. Through multivariate analysis, factors independently associated with persistent HD were identified, offering potential for improved risk stratification and procedural planning in CAS.

### 4.1 Anatomical and Procedural Influences on Hemodynamic Instability

Our findings indicate that multiple anatomical and procedural factors contribute to HD. Carotid bulb lesions were significantly more prevalent in patients who developed both intraoperative and persistent HD. This reinforces the pathophysiological relevance of baroreceptor density and sensitivity within this region. Prior studies similarly suggest that atherosclerotic involvement of the carotid sinus can enhance baroreceptor stimulation and predispose to HD [8, 10]. Calcified plaques were also more frequently observed in intraoperative and persistent HD groups, suggesting that plaque morphology may be associated with altered baroreflex activation rather than directly determining hemodynamic outcomes. It has been hypothesized that eccentric or heavily calcified lesions may increase procedural complexity and local mechanical stress at the stent site, potentially modulating baroreflex-mediated responses during stent expansion [7, 18]. Although calcified plaques were more frequently observed in patients without persistent hemodynamic depression, this finding should be interpreted with caution. Heavily calcified plaques may confer increased arterial rigidity, potentially limiting vessel deformation and reducing mechanical stimulation of the carotid sinus baroreceptors during stent expansion. However, despite this apparent univariate association, plaque calcification did not remain an independent predictor of persistent hemodynamic depression in multivariate analysis, suggesting that its effect is likely secondary to anatomical factors such as carotid bulb involvement and stent geometry. However, in our cohort, no significant differences in predilatation or postdilatation balloon diameters were observed between patients with and without HD. These findings suggest that balloon inflation strategy alone may not be a dominant determinant of HD development.

Collectively, our results highlight that HD development is multifactorial and should not be attributed solely to stent geometry. Lesion location and plaque morphology appear to be more relevant anatomical contributors, whereas procedural variables such as balloon sizing did not demonstrate a significant association with HD in our analysis.

### 4.2 Stent Type and Its Association with Persistent Hemodynamic Depression: Comparison with the Literature

In our study, the incidence of persistent HD was significantly higher among patients who received SCS compared to those who received TCS. Multivariate logistic regression analysis also identified the use of a straight stent as independently associated with persistent HD. This finding supports the hypothesis that stent geometry, particularly poor conformity to the native carotid anatomy, may exert increased mechanical pressure on the carotid sinus baroreceptors, potentially contributing to prolonged hemodynamic instability.

Although literature directly evaluating this relationship is limited, several key studies have reported consistent findings. Diehm *et al*. (2008) investigated the influence of stent design on HD and found that rigid nitinol stents were associated with higher rates of hemodynamic complications compared to more flexible designs [15]. Similarly, Csobay-Novák *et al*. (2015) demonstrated that stent selection significantly influenced the incidence of persistent HD, with tapered stents being associated with fewer adverse hemodynamic responses [16].​

A larger-scale study by Gupta *et al*. (2006) examined the predictors and consequences of HD following CAS and highlighted the importance of both technical and anatomical variables, although it did not focus specifically on stent design [6]. Conversely, AbuRahma *et al*. (2017) reported no significant association between stent type and HD development. However, in that study, HD was assessed as a general category without differentiating persistent HD, which may account for the discrepancy [17].​

Further supporting our findings, Liu *et al*. (2018) conducted a prospective study comparing SCS and TCS in patients with symptomatic carotid artery stenosis [19]. Their results indicated that TCS was associated with more favorable short-term outcomes, including a lower incidence of periprocedural complications, compared to SCS. Although the study did not specifically focus on persistent HD, the findings suggest that stent geometry may influence procedural tolerance and early complications.​

Differences among studies likely stem from heterogeneity in HD definitions, stent platforms, procedural protocols, and patient selection criteria. Moreover, persistent HD rates have been reported to range from 11% to 35%, mainly due to variations in definitions and measurements [10]. In addition, newer approaches such as TCAR are gaining clinical attention-TCAR has emerged as a promising minimally invasive option with favorable perioperative stroke profiles in high-risk patients, which may further influence future interpretations of HD risk; however, our study focuses explicitly on conventional CAS [20]. Our study contributes to the existing body of evidence by treating persistent HD not merely as a transient intraoperative finding, but as a clinically relevant endpoint, and by evaluating the role of stent geometry in its development through multivariate analysis.

### 4.3 Clinical Relevance of Persistent Hemodynamic Depression

In our study, persistent HD-defined as bradycardia and/or hypotension lasting beyond 24 hours despite medical treatment-was observed in 28 patients (25.7%). While transient forms of HD are often clinically tolerable, persistent HD has been associated with sustained cerebral hypoperfusion and has been linked to adverse clinical outcomes, particularly in elderly patients and those with significant comorbidities. Previous studies have indicated that prolonged hemodynamic instability following CAS may increase the risk of stroke, myocardial ischemia, and prolonged hospitalization [21, 22]. We did not observe statistically significant associations between persistent HD and major adverse events (stroke, TIA, restenosis, or mortality) during the 12-month follow-up. However, this may reflect a limited sample size rather than a lack of clinical relevance. Patients with persistent HD require closer hemodynamic monitoring and supportive care, potentially leading to increased healthcare utilization.

There is a growing consensus in the literature that HD should not be regarded as a benign or transient side effect but rather as a clinically meaningful variable with potential prognostic implications. Several studies have highlighted the association between sustained HD and worsened clinical outcomes [6, 19, 21]. While our study did not demonstrate a direct link between persistent HD and long-term morbidity, it underscores the clinical relevance of this condition. It aligns with prior reports advocating careful perioperative hemodynamic management following CAS.

### 4.4 Implications for Clinical Practice

Our results suggest that stent geometry and anatomical risk factors warrant consideration in procedural planning. The higher incidence of persistent HD in straight stents underscores the importance of considering devices that better align with the vascular anatomy. Carotid bulb involvement, also identified as an independent predictor, highlights the importance of individualized risk stratification, especially in elderly patients or those with cardiovascular comorbidities.

Current clinical practice guidelines for carotid artery stenting primarily focus on patient selection, degree of stenosis, and periprocedural management, while providing limited guidance on stent geometry selection. Our findings are consistent with existing recommendations emphasizing individualized procedural planning and anatomical considerations, particularly in patients with carotid bulb involvement. Rather than contradicting established CAS guidelines, our results extend current practice by highlighting stent geometry as an additional procedural factor that may influence the risk of persistent hemodynamic depression and warrant consideration during device selection [23].

From a practical standpoint, mitigation strategies such as temporarily withholding antihypertensive medications in the immediate preprocedural period, cautious postoperative reintroduction after hemodynamic stabilization, and optimized intravascular volume management may help reduce the risk of prolonged hemodynamic instability in high-risk patients. In our clinical practice, selected high-risk patients undergo continuous heart rate and blood pressure monitoring for at least 48 hours after carotid artery stenting, with readiness for intravenous fluid administration, vasopressor support, and temporary pacing when necessary, consistent with contemporary guideline-based recommendations for periprocedural blood pressure management during carotid interventions [5].

Importantly, these observations should be interpreted as hypothesis-generating and complementary to existing carotid artery stenting guidelines. While current recommendations appropriately emphasize patient selection and procedural safety, our results highlight procedural nuances that may inform individualized decision-making rather than serve as prescriptive guideline-level recommendations.

### 4.5 Study Limitations

This study has several limitations. First, its retrospective design introduces potential biases, particularly regarding the documentation and evaluation of hemodynamic parameters. Procedural and postprocedural management strategies may have varied according to clinical judgment and operator experience, which could have influenced the occurrence and management of hemodynamic depression.

Second, stent selection in this cohort was based on operator preference rather than randomization. Although this reflects real-world clinical practice, it may introduce selection bias and limit the ability to draw causal inferences regarding the impact of stent geometry on persistent hemodynamic depression.

Third, the sample size is relatively limited, especially in the subgroup of patients who developed persistent hemodynamic depression. This constraint may have reduced the statistical power to detect associations with long-term clinical outcomes, and some potentially relevant factors may not have reached statistical significance.

Finally, the follow-up period of this study was limited to 12 months. Longer-term, prospective, multicenter studies with extended follow-up are warranted to better define the neurological and cardiovascular consequences of persistent hemodynamic depression and to improve the generalizability of these findings to broader patient populations and procedural settings.

## CONCLUSION

Our findings suggest that persistent HD is neither rare nor clinically negligible following carotid artery stenting. The use of straight stents and the presence of carotid bulb lesions were identified as independent predictors of persistent HD. Given the potential implications for cerebral perfusion and clinical outcomes, preprocedural risk stratification and careful stent selection-favoring tapered designs in anatomically appropriate cases-may help minimize hemodynamic instability. Further prospective studies are warranted to validate these findings and explore long-term outcomes in larger patient populations.

## Figures and Tables

**Fig. (2):**
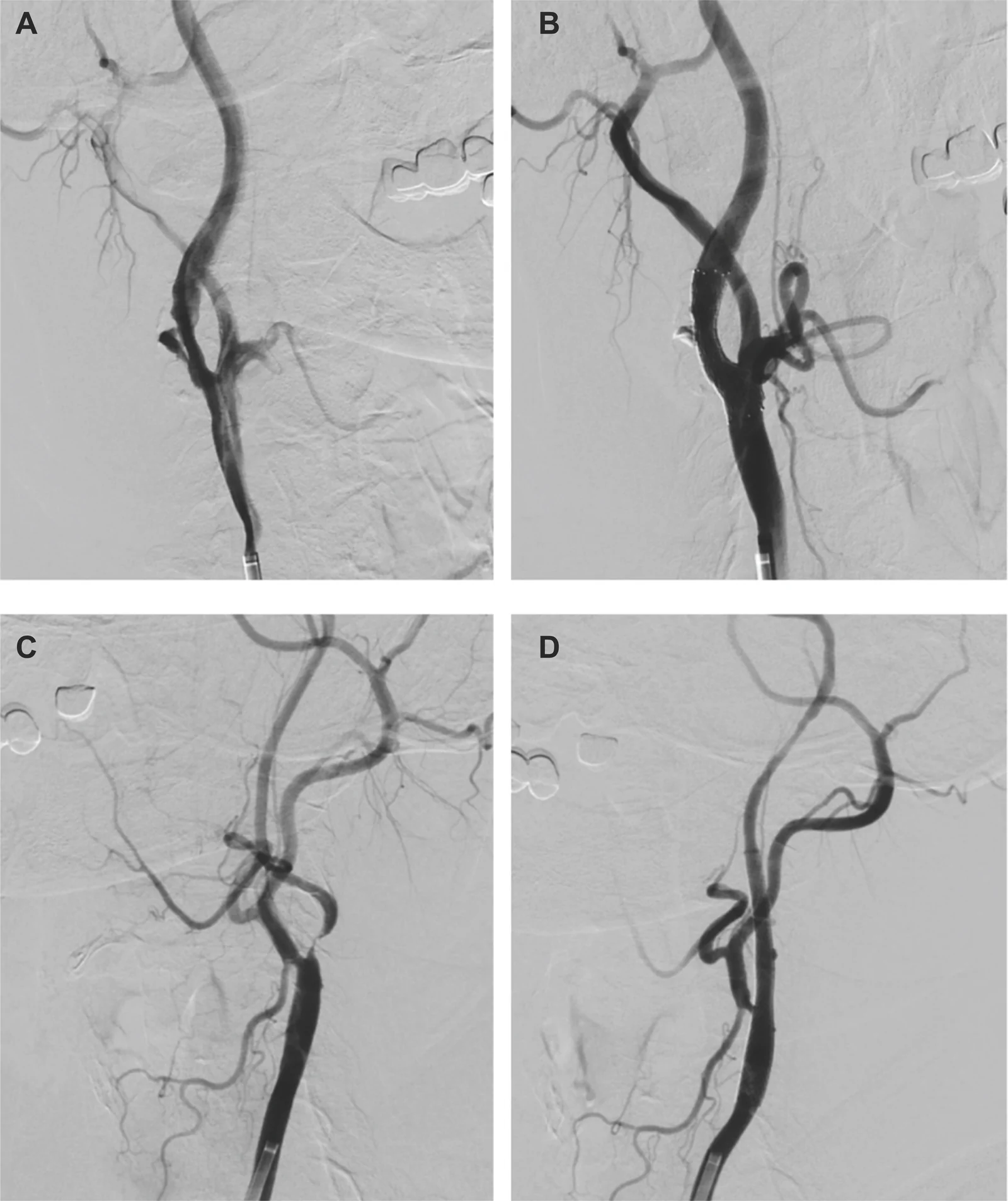
DSA images demonstrating pre- and post-procedural outcomes of tapered (**A**-**B**) and straight (**C**-**D**) stent implantation in carotid artery stenting.

**Table 1:** Patient characteristics stratified by periprocedural and persistent hemodynamic ınstability following CAS.

		**HD**		**Persistent**	**HD**	
	**Yes**	**No**	**p value**	**Yes**	**No**	***p*-value**
	**(n=44)**	**(n=65)**		**(n=28)**	**(n=81)**	
Mean age (y)	71.3± 6.1	71±6.7	0.615	71.1 ± 7.0	71,12±7.7	0.654
Hypertension n(%)	33(75.0)	51(78.5)	0.673	23(82.1)	61(75.3)	0.458
Diabetes mellitus n(%)	16(36.4)	24(36.9)	0.953	12(42.9)	32(39.5)	0.755
Smoking n(%)	29(65.9)	52(80.0)	0.099	24(85.7)	60(74.1)	0.207
Previous CAD n(%)	9(20.5)	18(27.7)	0.390	7(25.0)	15(18.5)	0.461
Stroke history n(%)	12(27.3)	24(36.9)	0.293	9(32.1)	22(27.2)	0.614
Symptomatic carotid stenosis n(%)	21(47.7)	42(64.6)	0.080	15(53.6)	42(51.9)	0.875
**Lesion involves carotid bulb n(%)**	**35(79.5)**	**40(61.5)**	**0.047**	**27(96.4)**	**46(56.8)**	**0.001**
**Calcified plaque n(%)**	**12(27.3)**	**59(90.8)**	**0.001**	**7(25.0)**	**63(77.8)**	**0.001**
ICA radius (mm, mean±SD)	1.89±0.27	1.88±0.23	0.662	1.82±0.78	1.89±0.81	0.877
Stent diameter (mm, mean±SD)	7.3±1.1	6.9±1.2	0.435	7.1±0.8	7.0±0.9	0.564
Predilation balloon diameter (mm, mean±SD)	3.3±0.6	3.1±0.8	0.103	3.7±0.8	3.5±1.0	0.873
Postdilation balloon diameter(mm mean±SD)	6.1±0.4	5.8±0.9	0.287	6.2±0.7	5.8±1.0	0.521
Preoperative systolic blood pressure(mm-Hg)	161±18	165±16	0.731	165±15	159±19	0.486
Preoperative heart rate	68±11	69±14	0.145	71±10	70±14	0.223

**Note:** Statistically significant P values (*P* < 0.05) are shown in bold.

**Table 2:** Comparison of hemodynamic outcomes and procedural characteristics between straight and tapered carotid stents.

	**SCS** **(N=38)**	**TCS** **(N=70)**	***p*-value**
Mean stent diameter			
CCA (mm mean±SD)	7.88±0.76	8.65±0.45	
ICA (mm mean±SD)	7.88±0.76	6.43±0.69	<0.001
Predilation n(%)	5(13.2)	11(15.7)	0.88
Postdilation n(%)	12(31.6)	31(44.3)	0.31
**Perioperative hemodynamic depression n(%)**	**19(50)**	**25(35.7)**	**<0.001**
Bradycardia only n(%)	6(15.8)	10(14.3)	0.34
Hypotension only n(%)	3(7.9)	4(5.7)	0.58
**Both n(%)**	**10(26.3)**	**11(15.7)**	**<0.001**
**Postoperative hemodynamic depression n(%)**	**15(39.5)**	**13(18.6)**	**<0.001**
**Bradycardia only n(%)**	**4(10.5)**	**4(5.7)**	**0.02**
Hypotension only n(%)	2(5.3)	3(4.3)	0.64
**Both n(%)**	**9(23.7)**	**6(8.6)**	**<0.001**

**Note:** Statistically significant *P* values (*P* < 0.05) are shown in bold.

**Table 3:** Multivariate logistic regression analysis of predictors for persistent hemodynamic depression.

**Variable**	**OR**	**95% CI**	***p-*value**
**Straight stenting (*vs.* tapered)**	**1.446**	**1.041-2.018**	**0.03**
**The lesion involves the carotid bulb**	**1.490**	**1.102-2.015**	**0.01**
Calcified plaque	1.345	0.642-5.259	0.67

**Note:** Statistically significant P values (*P* < 0.05) are shown in bold.

**Table 4:** Mid-term clinical outcomes and procedural complications in SCS *vs.* TCS Groups.

	SCS (N=38)	TCS (N=70)	*p-*value
Total stroke n(%)	1(2.6)	2(2.8)	0.82
Transient ischemic attack n(%)	2(5.2)	2(2.8)	0.47
Restenosis n(%)	0(0)	2(2.8)	0.58
Occlusion n(%)	1(2.6)	0(0)	0.66
Myocardial infarction n(%)	1(2.6)	0(0)	0.73
Pseudoaneurysm or hematoma n(%)	2(5.2)	1(1.4)	0.38
Death n(%)	0(0)	1(1.4)	0.63

## Data Availability

The datasets used and/or analyzed during the current study are available from the corresponding author [Y.Y] on reasonable request.

## LIST OF ABBREVIATIONS

CAS: Carotid Artery Stenting
HD: Hemodynamic Depression
TIA: Transient Ischemic Attacks
CEA: Carotid Endarterectomy
ICA: Internal Carotid Artery
CCA: Common Carotid Artery
TCAR: Transcarotid Artery Revascularization
DSA: Digital Subtraction Angiography
NASCET: North American Symptomatic Carotid Endarterectomy Trial
ACT: Activated Clotting Time
SCS: Straight Cell Stents
TCS: Tapered Cell Stents

## STANDARDS OF REPORTING

STROBE guidelines were followed.
